# Youthful insight: Nitrogen sequestration in larvae provides clues to coral bleaching

**DOI:** 10.1371/journal.pbio.3002890

**Published:** 2024-11-13

**Authors:** Christian R. Voolstra

**Affiliations:** Department of Biology, University of Konstanz, Konstanz, Germany

## Abstract

Impaired nutrient cycling under thermal stress foregoes coral bleaching, the loss of symbiotic algae. This Primer explores a new study in PLOS Biology which sheds light on how coral larvae avoid bleaching through nitrogen sequestration to uphold glucose translocation from their algal symbionts.

## Teaser

Impaired nutrient cycling under thermal stress foregoes coral bleaching, the loss of symbiotic algae. A new study in *PLOS Biology* sheds light on how coral larvae avoid bleaching through nitrogen sequestration to uphold glucose translocation from their algal symbionts.

Coral bleaching triggered by ocean warming is decimating coral reefs globally [1]. These iconic ecosystems are marine biodiversity hotspots and support the livelihood of close to a billion people [2]. The importance of coral reefs highlights the urgent need to elucidate the mechanisms behind coral bleaching, accelerating the development of strategies to mitigate the effects of thermal stress [3,4]. Despite the description of coral bleaching almost 100 years ago [5], we continue to make new discoveries about the cellular phenomena that underlie the loss of endosymbiotic photosynthetic algae from their coral hosts. Although reactive oxygen species (ROS) production induced by stress has been postulated as the primary culprit in triggering bleaching by means of oxidative damage [6], more recent studies argue that carbon-nitrogen exchange is a core process regulating symbiotic stability, with nitrogen-limitation by the host being an important mechanism for maintaining carbon translocation of the symbiont [7–9]. However, it was unclear how coral larvae counter thermal stress to avoid compromising the integrity of the algal symbiosis in this sensitive early life stage.

The recent study by Huffmyer and colleagues [10] in *PLOS Biology* sheds light on this by exposing symbiotic coral larvae to thermal stress and assessing their rates of photosynthesis, respiration, and metabolic exchange. Counter to expectation, the authors could show that the coral larvae avoided bleaching under thermal stress, which may provide important clues to inform coral bleaching at large. By limiting their own carbon metabolism and bolstering their nitrogen sequestration, coral larvae maintained algal photosynthetic rates and translocation of carbon, the hallmarks of a stable functioning coral–algal symbiosis. In other words, coral larvae compromised their own well-being to comfort their algal symbionts, keep them happy, and avoid cutting their symbiotic lifeline—short-term pains for long-term gains.

It is important to keep in mind that the study was conducted on larvae of *Montipora capitata*, a species known to be thermally resilient, and that the stress was relatively short term (3 days). This would make it interesting to pursue dynamics under longer-term stress regimes and to explore how larvae from other species may differ from the response in future studies. Nevertheless, it is remarkable that a temperature stress of +2.5°C did not impact larval survivorship, although reduced respiration rates indicative of metabolic depression became apparent. At present, it is unclear to what extent metabolic depression is cause or consequence of the observed pattern, i.e., the result of investing energy in nitrogen sequestration versus its employment as a mechanism for conserving energy. Counter to the former is the observation that the glucose pool size actually increased in coral larvae, suggesting that extra energy is available, but that cellular pathways besides glycolysis that maintain symbiotic homeostasis might be prioritized. Indeed, the findings simply further argue that symbiotic life is a delicate balance of giving and taking, involving a finely regulated nutrient balance, and that not everything is peachy at all times as put forward recently [11].

In sum, the study by Huffmyer and colleagues [10] provides further support that a carbon-nitrogen feedback loop underlies a stable coral–algal symbiosis and advances this notion by showing its general applicability across life stages (i.e., both larval and adult coral). Importantly, besides confirming the employment of the glutamine synthetase (GS) and glutamate dehydrogenase (GHD) pathways to regulate nitrogen availability, the authors found further evidence for a role of the urea cycle as an additional means to sequester nitrogenous metabolic waste. The authors also identified a putative novel mechanism for nitrogen sequestration: the generation of Arg-Gln dipeptides, which has been previously observed in adult corals [12]. It may be due to this that coral larvae were observed to increase their ammonium assimilation without a concurrent increase in carbon metabolic rates as described for adult corals, thus potentially de-coupling the burning of carbon fuel to the limiting of nitrogen availability.

The larval life stage is typically characterized as a critical phase, marking the transition from a free-swimming form to a settled juvenile coral. Larvae are crucial for the recovery and resilience of coral reefs by contributing to the replenishment of coral communities and biodiversity. Thus, understanding their response to thermal stress is important to project the future trajectory of coral. As always, new questions appear in pursuit of answering existing ones. It is compelling how symbiotic stability in larvae underlies the same principles as in adult corals, but with new twists that may ultimately inform our ability to counter the devastating impacts of thermal stress. For instance, limiting nitrogen availability (through environmental or biotic control) to the algal symbiont may stabilize the coral–algal symbiosis under thermal stress. One thing is for certain: the findings on larvae by Huffmyer and colleagues [10] just highlight the importance of harboring algal symbionts as the coral way of life—in happiness and adversity.
